# Dynamic changes of pulmonary function and immune function in children with mycoplasma pneumonia of different severity and their predictive value for disease prognosis: a retrospective cohort study

**DOI:** 10.3389/fmed.2025.1624256

**Published:** 2025-08-26

**Authors:** Zheng Liu, Wen Deng, Wenlin Xu, Linlin Ye, Zhihui Rao

**Affiliations:** ^1^Department of Respiratory, Children’s Hospital of Chongqing Medical University, Chongqing, China; ^2^Ministry of Education Key Laboratory of Child Development and Disorders, Children’s Hospital of Chongqing Medical University, Chongqing, China; ^3^National Clinical Research Center for Child Health and Disorders, Children’s Hospital of Chongqing Medical University, Chongqing, China; ^4^China International Science and Technology Cooperation base of Child development and Critical Disorders, Children’s Hospital of Chongqing Medical University, Chongqing, China; ^5^Chongqing Key Laboratory of Pediatrics, Children’s Hospital of Chongqing Medical University, Chongqing, China; ^6^Department of Pediatric, Jiangxi Children’s Medical Center, Nanchang, Jiangxi, China

**Keywords:** mycoplasma pneumonia, pulmonary function, immune function, diagnostic value, prognostic evaluation

## Abstract

**Purpose:**

To explore the dynamic changes in pulmonary and immune function among children with Mycoplasma pneumoniae pneumonia (MPP) and evaluate their value in disease severity stratification and prognosis prediction.

**Method:**

A retrospective cohort of 225 pediatric patients with varying degrees of MPP severity and disease course was analyzed. Lung function and immunological indices were measured and compared across groups.

**Results:**

Children with MPP exhibited significant impairments in pulmonary function and alterations in immune profiles compared to controls. These changes were associated with both disease severity and recovery status.

**Conclusion:**

Pulmonary and immune function markers may serve as useful indicators for assessing severity and recovery in pediatric MPP. Their clinical application could improve individualized management strategies.

## 1 Introduction

Mycoplasma pneumoniae (MP) is an atypical pathogen, sharing characteristics of both bacteria and viruses. The incidence of Mycoplasma pneumoniae pneumonia (MPP) comprises approximately 10%–40% of community-acquired pneumonia cases, with outbreaks occurring every 3–7 years (1, 2). The respiratory tract is the primary site of MP infection, with pneumonia occurring in 3%–10% of infected individuals. The disease course is typically benign, often asymptomatic or resolving without specific treatment (3). However, some children with MPP require treatment and may present challenges, although severe infections are rare, primarily affecting children aged 5–15 years (4). In recent years, there has been an increasing incidence of MPP among infants (5). Researchers are increasingly focusing on the pathogenesis, clinical symptoms, and laboratory indicators of MPP (6). MP infection can cause airway hyperreactivity, pulmonary dysfunction, and immune system disruption. Unlike non-MP infections, MP-related illnesses often persist and recur, with some patients suffering from delayed diagnosis or inadequate treatment due to a lack of awareness and prognostic insight. This can lead to long-term complications such as chronic cough, asthma, bronchiolitis obliterans, and permanent lung damage, severely affecting children’s quality of life and placing a burden on families (7). among the diagnostic and therapeutic challenges of MP infections in children, there are also the extra-pulmonary manifestations and diseases (8). Emerging evidence suggests that MP infection is significantly associated with the development or exacerbation of asthma and long-term lung function decline, especially in predisposed children (9). Additionally, MP may disrupt immune balance by altering T lymphocyte subset profiles, contributing to both acute inflammation and chronic airway remodeling (10). Pulmonary function examination has the advantages of non-invasive, convenient, and simple operation, which provides great help for clinical diagnosis and treatment (11). T lymphocyte subgroups are crucial immune cell populations in the human body, functioning both as effector cells of immune responses and as regulators of immune function. Among T lymphocyte subsets, CD3+ T cells serve as characteristic markers of mature T lymphocytes, while CD4+ T and CD8+ T lymphocytes are central to cellular immunity. The CD4+/CD8+ ratio reflects the body’s immune status, with a decreased ratio indicating suppressed cellular immune function (12). Based on previous studies, which have indicated that changes in lung function and immune function can, to some extent, reflect the progression of MPP (13), this study aims to further explore these changes in children with varying degrees of MPP severity. By analyzing lung function and immune function alterations, this research seeks to provide valuable insights for improving the diagnosis and prognosis of the disease. In addition, this study aims to address existing gaps in current knowledge by offering a more detailed understanding of how these physiological changes correlate with disease severity, which could contribute to more precise diagnostic and prognostic tools for MPP.

## 2 Materials and methods

### 2.1 Study design

This was a retrospective observational study conducted at the Children’s Hospital of Chongqing Medical University between January 2021 and January 2023. The study included both clinical and control participants and was designed to explore the relationship between MPP severity, lung function, and immune status in pediatric patients.

A total of 225 newly diagnosed and hospitalized children with confirmed MPP were enrolled consecutively. To eliminate confounding effects from prior intervention, all patients were assessed and grouped within 24 h of admission, and before the initiation of specific antimicrobial or immunomodulatory treatments.

**FIGURE 1:**
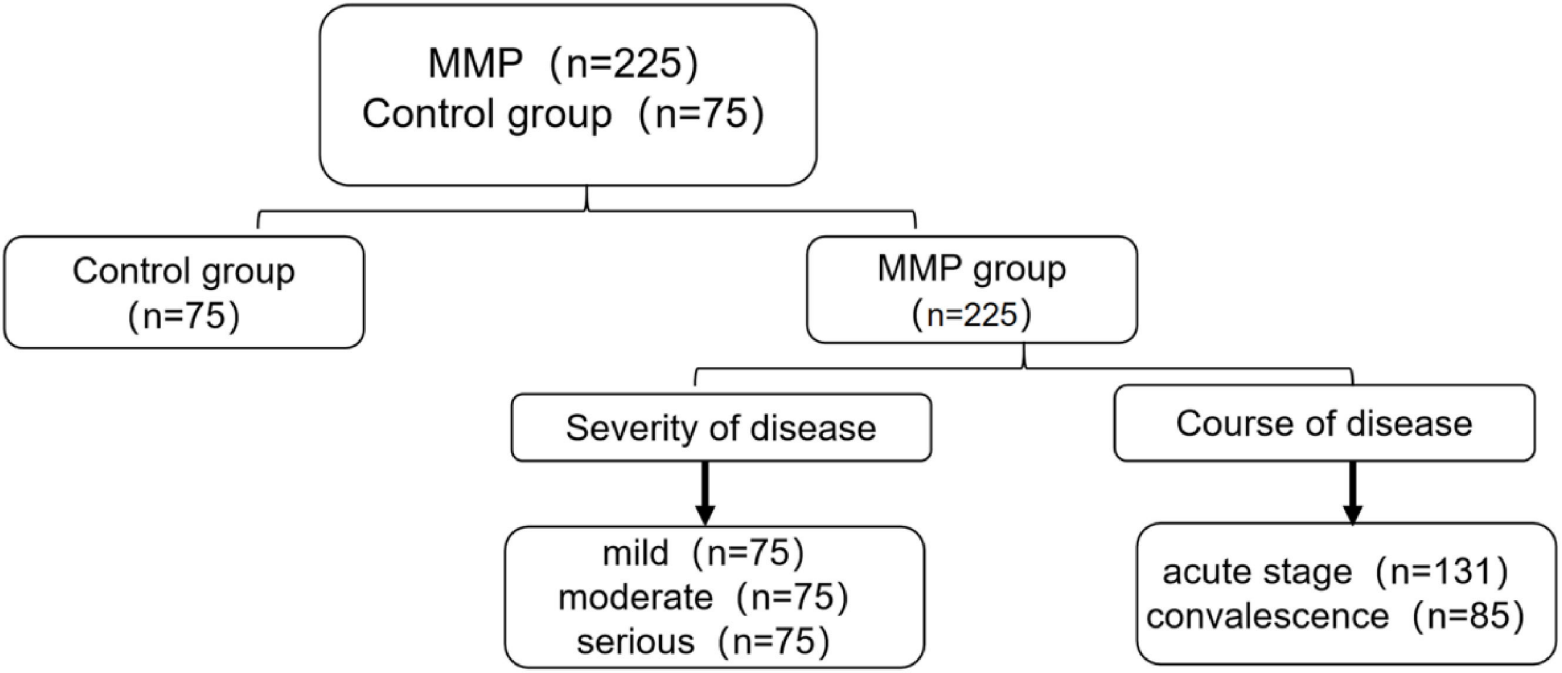
Research flow chart.

Furthermore, to analyze temporal changes in physiological parameters, the MPP group was divided into acute-phase (*n* = 131) and recovery-phase (*n* = 85) subsets, based on clinical progression and time of sample collection. The overall participant selection strategy, grouping logic, and analytical flow are illustrated in Figure 1 for clarity.

### 2.2 Clinical data

#### 2.2.1 Research objects

This retrospective study included four groups: children with mild (*n* = 75), moderate (*n* = 75), and severe MPP (*n* = 75), and healthy controls (*n* = 75). All MPP patients were newly diagnosed upon admission and had not received disease-specific treatment prior to enrollment. Grouping by severity was based on initial clinical and laboratory evaluation within 24 h of hospitalization.

To further investigate the disease course, the MPP group was divided into:

1. An acute-phase group consisting of patients in the symptomatic or peak inflammatory stage, with samples collected early in hospitalization;
2. A recovery-phase group composed of patients whose symptoms had improved significantly, with samples collected ≥14 days after symptom onset and after clinical stabilization.

All patients received standardized management according to institutional MPP guidelines following admission. Therefore, grouping by disease severity and disease stage was not affected by treatment heterogeneity, as seen in Supplementary Table 1.

Inclusion and exclusion criteria were consistently applied to ensure comparability between groups. Details of the participant distribution and grouping flow are shown in Figure 1.

#### 2.2.2 Diagnostic criteria

Diagnosis of MPP was made based on internationally recognized guidelines, incorporating clinical, radiological, and laboratory criteria, as follows:

A diagnosis was established when all the following three components were present:

1. Clinical criteria:

(a) Acute onset of respiratory symptoms, particularly persistent or paroxysmal dry cough lasting more than 3–5 days;

(b) With or without fever, and respiratory signs such as coarse breath sounds, wheezing, or rales on auscultation.

2. Radiologic criteria:

Chest imaging (X-ray or CT) showing findings consistent with atypical pneumonia, including interstitial infiltration, peribronchial thickening, patchy consolidation, or segmental/lobar opacity.

3. Laboratory/etiologic evidence:

(a) Positive Mycoplasma pneumoniae-specific IgM antibody titer≥1:160 by ELISA (EUROIMMUN, Germany), or

(b) PCR detection of MP-DNA from nasopharyngeal swabs or sputum (using ABI 7500 system, Applied Biosystems, USA), or

(c) Serologic evidence of a ≥ 4-fold rise in MP antibody titer between acute and convalescent serum samples taken≥14 days apart.

##### 2.2.2.1 Criteria for Assessing Disease Severity and Disease Progression Stage

Disease severity was assessed according to updated clinical guidelines and classified into three levels:

(1) Mild MPP

(a)    Fever duration: ≤3 days, body temperature < 38.5 °C
(b)    Cough: Mild, non-disruptive to daily activity or sleep
(c)    Respiratory status: No dyspnea or retraction signs
(d)    Imaging: Localized or unilateral patchy infiltrates, no consolidation or effusion
(e)    Laboratory findings: CRP 10–30 mg/L, WBC count 10–12 × 10^9^/L

(2) Moderate MPP

(a)    Fever duration: 4–6 days, temperature between 38.5 °C and 39.5 °C
(b)    Cough: Frequent, may interfere with rest or feeding
(c)    Respiratory status: Mild to moderate dyspnea, nasal flaring or mild chest retractions
(d)    Imaging: Bilateral or multilobar involvement, mild consolidation or small pleural effusion
(e)    Laboratory findings: CRP 31–60 mg/L, WBC count 13–15 × 10^9^/L

(3) Severe MPP

(a)    Fever duration: >6 days, temperature > 39.5 °C
(b)    Cough: Persistent, paroxysmal or exhausting
(c)    Respiratory status: Significant dyspnea, cyanosis, tachypnea, chest wall retractions, or hypoxia requiring oxygen support
(d)    Imaging: Diffuse infiltration, lobar consolidation, or moderate/severe pleural effusion
(e)    Laboratory findings: CRP > 60 mg/L, WBC > 15 × 10^9^/L, and elevated PCT/ESR
(f)    Disease progression was categorized as follows:

(4) Acute phase

(a)    Persistent respiratory symptoms (e.g., cough, dyspnea, fever)
(b)    Imaging shows active lung inflammation or progression of infiltrates
(c)    MP-specific IgM ≥ 1:160 or MP-DNA positive
(d)    Paired sera demonstrate ≥4-fold increase in antibody titer

(5) Recovery phase

(a)    Clinical improvement: fever resolved, cough reduced or absent, dyspnea relieved
(b)    Imaging shows significant absorption or resolution of lung lesions
(c)    MP-specific IgM ≤ 1:160, MP-DNA negative
(d)    Paired sera show ≥ 4-fold decrease in antibody titer compared to acute phase

In the diagnostic criteria for the acute phase, all four criteria (a-d) must be satisfied to establish a definitive diagnosis. In contrast, during the recovery phase, fulfilling three out of the four criteria is sufficient for diagnosis. To clearly distinguish the recovery phase from healthy controls, the recovery group still had minor or resolving radiological changes (e.g., residual shadows without new lesions), while control subjects exhibited no clinical symptoms and had completely normal radiological and immune profiles.

#### 2.2.3 Inclusion criteria

The inclusion criteria for the patient group were as follows: (1) meeting the relevant diagnostic criteria for MPP, all patients undergo MP DNA testing, Serum MP-Ab-IgM is positive or cold agglutination≥1:32; (2) aged between 3 and 12 years old; (3) absence of other infections within the past month; and (4) signed informed consent from the patients’ family members.

Exclusion criteria for the patient group were: (1) Multiplex PCR diagnosis was MPP complicated with other bacterial and viral infections; (2) presence of underlying pulmonary diseases (such as asthma, tuberculosis, tracheomalacia, congenital airway dysplasia), allergic diseases, or severe organ dysfunction or systemic diseases; (3) systemic use of glucocorticoids, immunomodulators, or leukotriene receptor antagonists within the month prior to admission; and (4) poor compliance or difficulty in cooperating with diagnostic and treatment procedures.

The inclusion criteria for the control group were: (1) having undergone physical examination at our hospital; (2) normal results on blood tests and pulmonary function tests; and (3) family members’ awareness and agreement to participate in the study.

Exclusion criteria for the control group were: (1) dropout from the study or participation in other research studies; and (2) poor compliance or difficulty in cooperating with diagnostic and treatment procedures.

#### 2.2.4 Sample size calculation

Based on conventional multivariable design rules (5–10 × number of predictors), with 6 main variables analyzed, a minimum sample size of 60 was calculated. To compensate for attrition, 25% inflation yielded 75 per group.

### 2.3 Detection method

#### 2.3.1 Detection of pulmonary function index

Pulmonary function was assessed using the Jaeger MasterScreen™ spirometer (CareFusion, Germany), operated in accordance with American Thoracic Society/European Respiratory Society (ATS/ERS) guidelines for pulmonary function testing in children.

Prior to testing, basic anthropometric data (height, weight) were recorded. Subjects were required to rest quietly for at least 30 min. Standardized instructions were provided through animated demonstrations and verbal explanations to ensure correct technique and reduce anxiety.

During the test, participants were seated upright with a neutral head position, biting the mouthpiece gently with sealed lips. At least three acceptable spirometry maneuvers were performed, with the highest values recorded for analysis.

The following parameters were measured:

(a)    Forced Vital Capacity (FVC, L);
(b)    Forced Expiratory Volume in 1 s (FEV1, L);
(c)    Peak Expiratory Flow (PEF, L/s).

Quality control was ensured by trained technicians, with daily device calibration using a 3.0 L syringe (CareFusion calibration syringe).

#### 2.3.2 Detection of immune function index

##### 2.3.2.1 Sample collection

(a)    Fasting venous blood (3 mL) was collected from each participant using sterile EDTA tubes (Vacutainer®, BD, USA).
(b)    For MPP patients:
(c)    Acute phase samples were collected within 24 h of admission;
(d)    Recovery phase samples were collected after resolution of symptoms and radiologic improvement.
(e)    For healthy controls, samples were obtained during routine physical examination.

Blood samples were centrifuged using a Hettich Rotina 380R refrigerated centrifuge (Germany) at 3000 rpm for 10 min (centrifugal radius: 8 cm). Plasma and cells were separated and stored at −80 °C until testing.

##### 2.3.2.2 Lymphocyte Immunophenotyping

(a)    Performed via flow cytometry using the BD FACSCanto™ II system (BD Biosciences, USA).
(b)    Monoclonal antibodies were sourced from BD Multitest™ CD3/CD4/CD8/CD45 reagent kit (BD Biosciences, USA).
(c)    Data analysis was conducted with FACSDiva™ software (BD Biosciences).

The following immune parameters were measured:

(a)    CD4+ T lymphocyte percentage (%);
(b)    CD8+ T lymphocyte percentage (%);
(c)    CD4+/CD8+ ratio.

#### 2.3.3 Mycoplasma pneumoniae serological and molecular testing

Serology:

(a)    MP-specific IgM antibody levels were determined using a commercial Enzyme-Linked Immunosorbent Assay (ELISA) kit (EUROIMMUN, Germany).
(b)    An IgM titer ≥ 1:160 was considered positive.

Molecular Detection:

(a)    MP-DNA was extracted from nasopharyngeal swabs using the QIAamp® DNA Mini Kit (QIAGEN, Germany).
(b)    Real-time PCR amplification was performed using the ABI 7500 Real-Time PCR System (Applied Biosystems, USA) with primers and probes targeting the P1 adhesin gene, following manufacturer protocols.

### 2.4 Observation index

#### 2.4.1 Diagnostic criteria

The gold standard of MPP diagnosis was based on the results of etiological examination. The critical value of lung function index diagnosis: FVC ≥ 80% is normal, 60% Murray 79% is mild injury, 40% Murray 59% is moderate injury, ≤39% is severe injury. FEV1 ≥ 80% is normal, 60% Murray 79% is mild injury, 40% Murray 59% is moderate injury, ≤39% is severe injury. PEF ≥ 80% is normal, 60% Murray 79% is mild injury, 40% Murray 59% is moderate injury, ≤39% is severe injury. >80% is normal lung function, and≤79% is decreased, that is, if any one of the different detection methods appears<80%, it is judged as injury.

The critical value of immune function index diagnosis: CD4+T: 25%–40%; CD8+T: 0%–30%; and calculate CD4+T/CD8+T: 1.2–2. If it is lower than the lower limit, it is judged as decreasing, and if it is higher than the upper limit, it is judged as increasing. That is, if any one of the different detection methods is positive, it is determined to be positive.

#### 2.4.2 Evaluation of diagnostic effect

Sensitivity, specificity, accuracy, positive predictive value, negative predictive value and Area Under Curve (AUC) were used to evaluate the diagnostic effect. AUC < 0.5 is considered as no diagnostic value, diagnostic accuracy between 0.5 and 0.7 is low, diagnostic accuracy between 0.7 and 0.9 is good, and AUC > 0.9 is considered as the highest diagnostic accuracy. Sensitivity, specificity, accuracy, positive predictive value and negative predictive value are calculated as follows: Sensitivity = $\frac{a}{a+c}\times 100\%$, specific degrees = $\frac{d}{b+d}\times 100$, accuracy = $\frac{a+d}{a+b+c+d}\times 100\%$, positive predictive value = $\frac{a}{a+b}\times 100\%$ and negative predictive value = $\frac{d}{c+d}\times 100\%$, among them, a method for the diagnosis of testing positive for the number of cases, b is the number of positive cases in the test method control group, c is the number of negative cases detected by the test method, and d is the number of negative cases in the test control group.

### 2.5 Statistical analysis

All data analyses were performed using SPSS version 23.0 (IBM Corp., Armonk, NY, USA). The normality of continuous variables was assessed using the Kolmogorov-Smirnov test. Normally distributed data were presented as mean ± standard deviation (SD) and analyzed using parametric tests; non-normally distributed data were expressed as median (interquartile range, IQR) and analyzed with non-parametric methods. Levene’s test was used to assess the homogeneity of variance. For between-group comparisons, independent sample *t*-tests (two groups) or one-way ANOVA with Tukey’s post-hoc test (multiple groups) were applied when assumptions were met; otherwise, Mann-Whitney U or Kruskal-Wallis *H* tests were used. Categorical variables were expressed as frequency (*n*) and percentage (%), and evaluated using the Chi-square test or Fisher’s exact test where applicable. Pearson correlation analysis was used to assess relationships between immune parameters and pulmonary function indices; Spearman correlation was used for non-parametric variables. Diagnostic efficacy was evaluated using ROC curve analysis, with calculation of area under the curve (AUC), sensitivity, specificity, accuracy, positive predictive value (PPV), and negative predictive value (NPV); comparisons between AUCs were performed using the Delong test. All statistical tests were two-tailed, and a *P*-value < 0.05 was considered statistically significant.

## 3 Results

### 3.1 Comparison of pulmonary function indexes between control group and MPP children with different severity and course of disease

**FIGURE 2:**
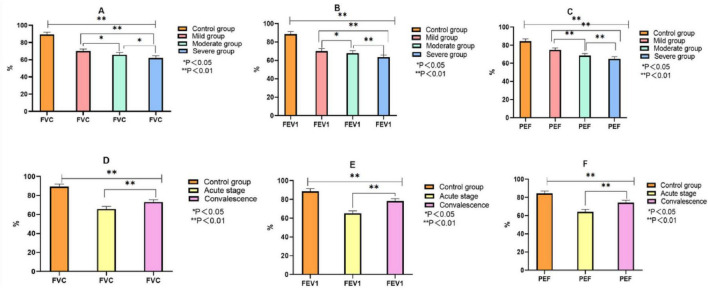
Comparison of pulmonary function indexes between control group and MPP children with different severity and course of disease. Note: **(A–C)** comparison of FVC, FEV1 and PEF between the control group and children with different severity of MMP. **(D–F)** The comparison of FVC, FEV1 and PEF between the control group and MMP children with different courses.

As illustrated in Figure 2, children with MPP exhibited significant reductions in pulmonary function compared to healthy controls. Specifically: The FVC, FEV1, and PEF values progressively decreased with increasing disease severity from mild to severe MPP. These reductions were statistically significant (*P* < 0.05), indicating greater impairment of pulmonary function in children with more severe disease. When comparing disease stages, children in the acute phase had significantly lower FVC, FEV1, and PEF than those in the recovery phase. This upward trend in pulmonary function during recovery was statistically significant (*P* < 0.05).

### 3.2 Comparison of immune indexes between control group and children with MPP of different severity and course of disease

**FIGURE 3:**
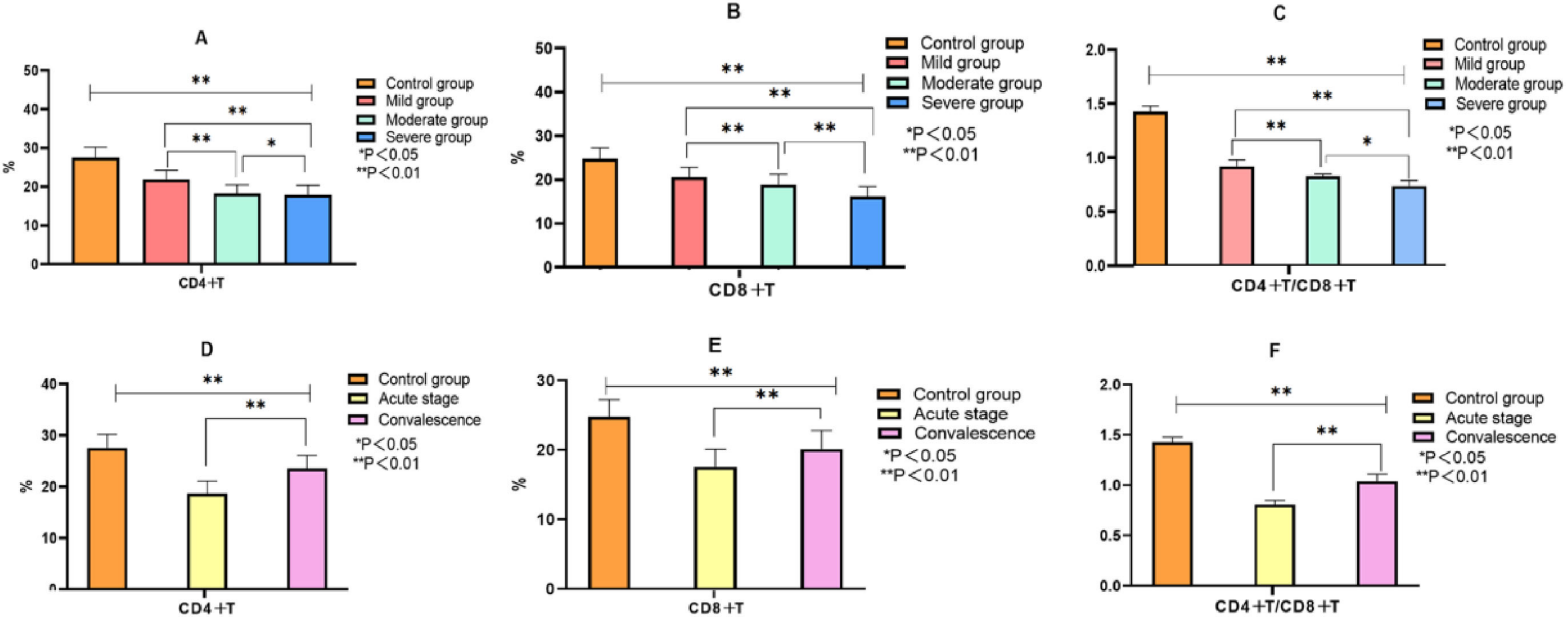
Comparison of immune indexes between the control group and MPP children with different severity and course of disease. Note: **(A–C)** Comparison of CD4+T, CD8+T, CD4+T/CD8+T between control group and MMP children with different severity; **(D–F)** Comparison of CD4+T, CD8+T, CD4+T/CD8+T between control group and MMP children with different disease course.

As presented in Figure 3, the immune profiles of children with MPP differed significantly from healthy controls and varied according to both disease severity and course: CD4^+^T and CD8^+^T cell percentages were significantly elevated in MPP children compared with the control group and showed a progressive increase with advancing disease severity from mild to severe cases (*P* < 0.05). Conversely, the CD4^+^/CD8^+^ ratio exhibited a significant decrease corresponding to increased severity (*P* < 0.05). During the transition from the acute to the recovery phase, both CD4^+^T and CD8^+^T cell percentages demonstrated a marked decline (*P* < 0.05), while the CD4^+^/CD8^+^ ratio showed a significant upward trend, indicating a gradual restoration of immune balance with disease resolution (*P* < 0.05).

### 3.3 Correlation between pulmonary function, immune index and MPP index

Pearson correlation analysis showed that FVC, FEV1, PEF, CD4^+^T, CD8^+^T and CD4^+^/CD8^+^ ratio were negatively correlated with the severity of MMP disease, and the data difference was statistically significant (*P* < 0.05). FVC, FEV1, PEF, CD4^+^T, CD8^+^T, CD4^+^/CD8^+^ ratio were positively correlated with the disease recovery of MMP, and the data difference was statistically significant (*P* < 0.05, Table 1).

**TABLE 1:** Correlation between pulmonary function, immune index and MPP index [*n*/%].

Variable	Severity of MMP		Recovery status of MMP	
	*r*	*P*	*r*	*P*
FVC	−0.523	<0.001	0.653	<0.001
FEV1	−0.556	<0.001	0.544	<0.001
PEF	−0.623	<0.001	0.485	<0.001
CD4+T	−0.692	<0.001	0.732	<0.001
CD8+T	−0.542	<0.001	0.672	<0.001
CD4^+^/CD8^+^ ratio	−0.634	<0.001	0.612	<0.001

### 3.4 Multivariate regression analysis of risk factors affecting the occurrence of MPP

Assign values to FVC, FEV1, PEF, CD4^+^T, CD8^+^T, CD4^+^/CD8^+^ ratio. If FVC, FEV1, PEF ≥ 80%, the value is 0; if<80%, the value is 1; CD4+T: 25–40% = 0, <25% = 1; CD8+T: 20–30% = 0, <20% = 1; CD4^+^/CD8^+^: <1.2 = 0, 1.2 –2 = 1. Table 2 showed that FVC, FEV1, PEF, CD4^+^T, CD8^+^T, CD4^+^/CD8^+^ were protective factors for MMP disease severity, and the data differences were statistically significant (*P* < 0.05). The results in Table 3 showed that FVC, FEV1, PEF, CD4^+^T, CD8^+^T and CD4^+^/CD8^+^ ratio are the protective factors for the recovery of MMP disease in acute stage, and the data difference is statistically significant (*P* < 0.05).

**TABLE 2:** Multivariate logistic regression analysis of pulmonary and immune function parameters predicting MPP severity.

Variable	*b*	S.E	Wald	*P*	OR	95%CI for OR
FVC	−0.982	0.232	17.916	0.000	0.375	0.238–0.590
FEV1	−0.984	0.223	19.471	0.000	0.374	0.241–0.579
PEF	−0.843	0.220	14.683	0.000	0.430	0.280–0.662
CD4^+^T	−0.842	0.384	4.808	0.028	0.431	0.203–0.915
CD8^+^T	−0.944	0.434	4.731	0.030	0.389	0.166–0.911
CD4^+^/CD8^+^ ratio	−1.434	0.232	38.205	0.000	0.238	0.151–0.376

**TABLE 3:** Multivariate logistic regression analysis of pulmonary and immune function parameters predicting MPP recovery status.

Variable	*b*	S.E	Wald	*P*	OR	95%CI for OR
FVC	−0.784	0.244	10.324	0.001	0.458	0.283–0.737
FEV1	−0.711	0.245	8.422	0.004	0.491	0.304–0.794
PEF	−0.783	0.383	4.180	0.041	0.457	0.216–0.968
CD4^+^T	−0.744	0.272	7.482	0.006	0.475	0.279–0.810
CD8^+^T	−0.692	0.233	8.821	0.003	0.501	0.317–0.790
CD4^+^/CD8^+^ ratio	−0.945	0.255	13.374	0.000	0.389	0.236–0.641

### 3.5 ROC curve of pulmonary function and immune index in the diagnosis of MPP with different severity and different course of disease

**FIGURE 4:**
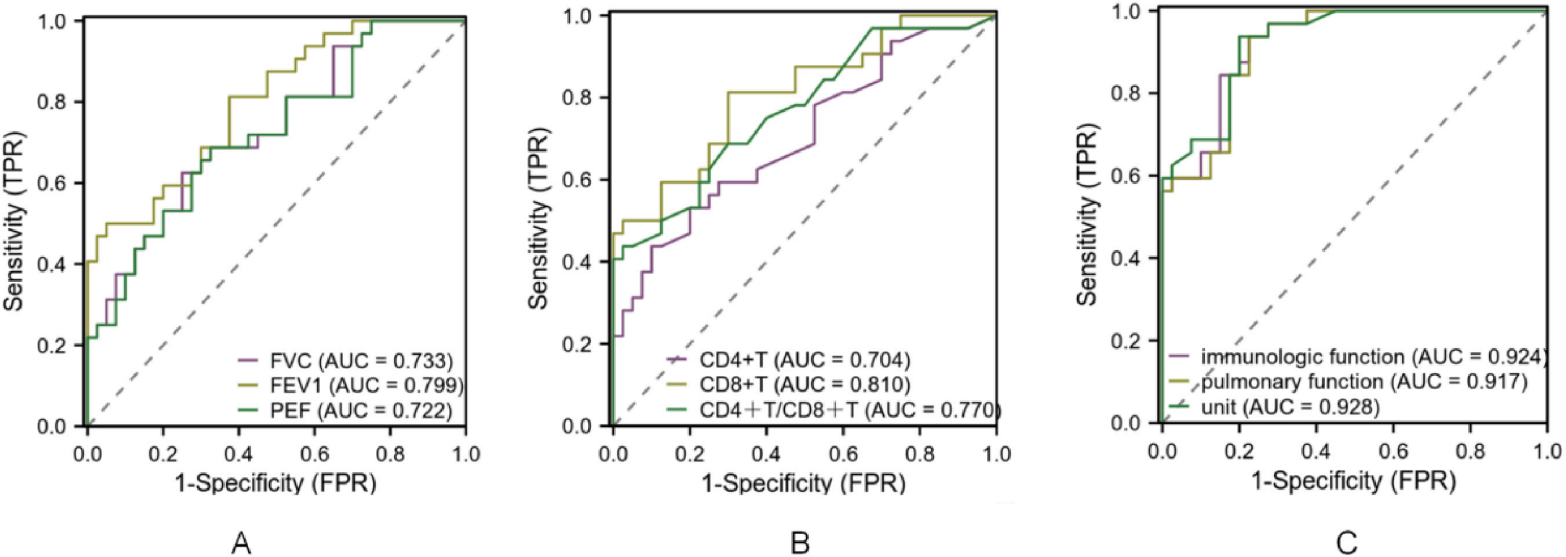
ROC curve of pulmonary function and immune index in the diagnosis of MPP with different severity. Note: **(A)** ROC curve of FVC, FEV1 and PEF for diagnosing MPP of different severity; **(B)** ROC curves of CD4+T, CD8+T, CD4+T/CD8+T for diagnosing MPP of different severity. **(C)** ROC curves of immunologic function and pulmonary function for diagnosing MPP of different severity.

**FIGURE 5:**
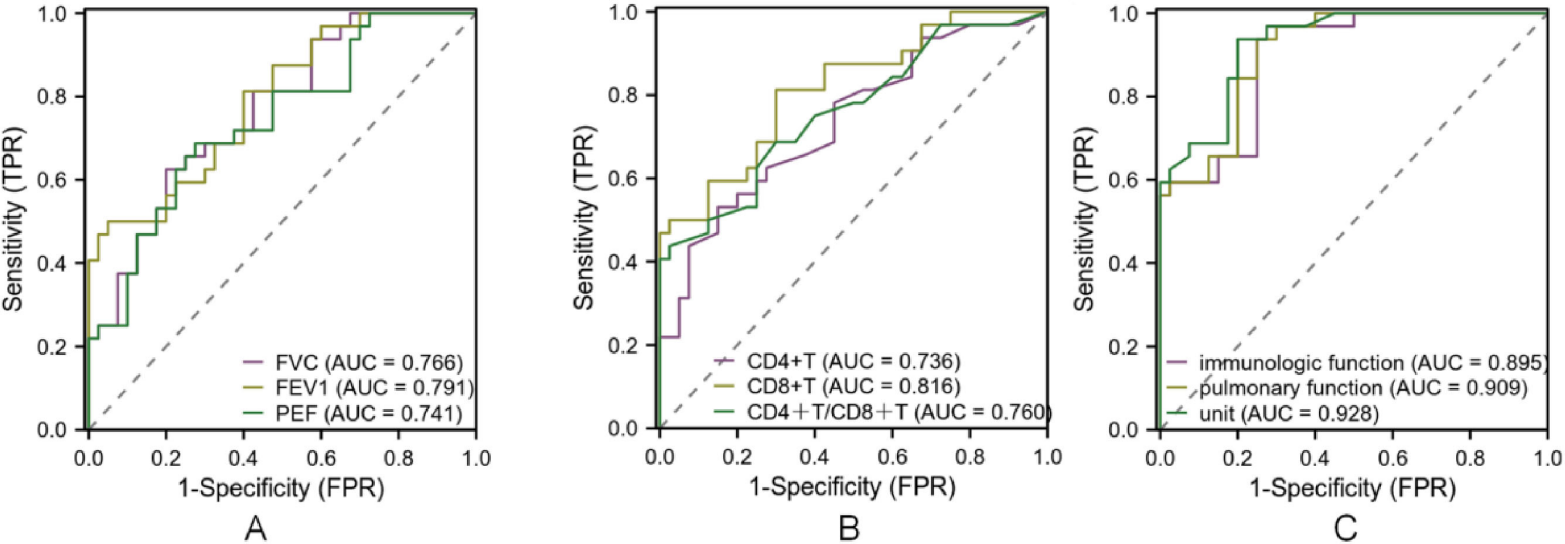
ROC curve of lung function and immune index in the diagnosis of MPP in different course of disease. Note: **(A)** ROC curve of FVC, FEV1 and PEF for diagnosing MPP of different disease course; **(B)** ROC curves of CD4+T, CD8+T, CD4+T/CD8+T in diagnosing MPP of different disease course; **(C)**: ROC curve of lung function index, immune function index and combined diagnosis of MPP of different disease course.

The diagnostic performance of pulmonary function and immune indices for identifying disease severity and distinguishing between disease phases was evaluated. As shown in Table 4, sensitivity, specificity, accuracy, and predictive values demonstrated acceptable diagnostic ability. ROC analysis further confirmed these findings, with AUC values indicating good diagnostic accuracy (Figure 4). For disease severity, FVC, FEV1, and PEF showed sensitivity values ranging from 0.625 to 0.687 and specificity ranging from 0.675 to 0.950, with AUC values of 0.733, 0.799, and 0.722, respectively. Immune indices such as CD4^+^T and CD8^+^T demonstrated sensitivity and specificity ranging from 0.437 to 0.812 and 0.700 to 0.975, respectively, with AUC values of 0.704 to 0.810. The combined pulmonary function indices had a sensitivity of 0.937 and specificity of 0.774. Regarding disease course, FVC, FEV1, and PEF demonstrated sensitivity ranging from 0.562 to 0.684 and specificity from 0.725 to 0.924, with AUC values between 0.741 and 0.791. For immune indices, CD4^+^T, CD8^+^T, and CD4^+^/CD8^+^ ratio showed sensitivity values from 0.531 to 0.812 and specificity ranging from 0.700 to 0.922. The combined pulmonary function indices for disease course yielded a sensitivity of 0.923 and specificity of 0.734. All AUC values indicated acceptable to good diagnostic accuracy (*P* < 0.05; Figure 5 and Table 5).

**TABLE 4:** Diagnostic performance of pulmonary and immune parameters in MPP severity stratification.

Index	Sensitivity degree	Specificity degree	Accuracy	Positive predictive value	Negative predictive value
FVC	0.625	0.750	0.694	0.666	0.714
FEV1	0.500	0.950	0.750	0.888	0.703
PEF	0.687	0.675	0.680	0.625	0.729
CD4+T	0.437	0.900	0.694	0.777	0.666
CD8+T	0.812	0.700	0.750	0.684	0.823
CD4^+^/CD8^+^ ratio	0.437	0.975	0.736	0.933	0.684

**TABLE 5:** Diagnostic performance of pulmonary and immune parameters in MPP course stratification (Acute vs. Recovery).

Index	Sensitivity degree	Specificity degree	Accuracy	Positive predictive value	Negative predictive value
FVC	0.625	0.800	0.722	0.714	0.727
FEV1	0.562	0.924	0.764	0.824	0.723
PEF	0.684	0.725	0.708	0.763	0.743
CD4+T	0.531	0.850	0.708	0.739	0.672
CD8+T	0.812	0.700	0.750	0.684	0.812
CD4^+^/CD8^+^ ratio	0.545	0.922	0.749	0.924	0.681

## 4 Discussion

Recent clinical observations suggest that both younger and older children tend to present with more severe manifestations of MPP (14). The diversity in symptoms–ranging from irritant cough and fever to wheezing – highlights the variable clinical course in pediatric populations (15, 16). These may include persistent cough, wheezing, fever, or even extrapulmonary complications. Such variability underlines the importance of evaluating pulmonary function in both acute and recovery phases. Our study found that FVC, FEV1, and PEF declined significantly with increasing MPP severity and partially recovered during convalescence, although they remained below control levels. This supports previous findings that lung function impairment correlates closely with disease severity and stage (17). These trends suggest that pulmonary injury begins early in the course of infection and necessitates ongoing clinical attention during recovery to prevent long-term sequelae.

Mycoplasma infection disturbs immune homeostasis, particularly the CD4^+^/CD8^+^ T-cell axis. CD4^+^ T cells assist in activating immune responses, while CD8^+^ T cells are cytotoxic and may promote tissue damage when dysregulated (18). Previous studies have demonstrated a decrease in CD4^+^ T cell proportion and CD4^+^/CD8^+^ ratio, and an increase in CD8^+^ T cells in severe MPP casest (19–21). Our results were consistent with these reports. In children with increasing MPP severity, we observed higher CD8^+^ counts, lower CD4^+^/CD8^+^ ratios, and persistent immune imbalance even into the recovery phase. This dysregulation may impair effective clearance of MP and exacerbate tissue damage, contributing to a vicious inflammatory cycle (22, 23).

Pearson correlation and logistic regression analyses further confirmed that declines in pulmonary indices (FVC, FEV1, PEF) and altered immune markers (CD4^+^, CD8^+^, CD4^+^/CD8^+^) were significantly associated with both MPP severity and recovery.

Notably, these variables were identified as independent protective factors, suggesting their potential utility in clinical risk stratification. The reduction in pulmonary function likely reflects inflammatory airway obstruction, while immune marker changes indicate compromised immune regulation (24). ROC curve analyses also demonstrated strong predictive performance, with AUC values for combined lung function and immune markers exceeding 0.75 in most comparisons. These findings suggest that integrating pulmonary and immune biomarkers can enhance diagnostic precision and disease monitoring.

Given the chronic and relapsing nature of MPP in some pediatric cases, dynamic monitoring of lung and immune function is critical. The combined assessment allows for real-time evaluation of disease activity and may guide treatment decisions, especially in severe cases. This approach supports individualized management, early identification of at-risk patients, and optimization of follow-up strategies. Therefore, incorporating FVC, FEV1, PEF, CD4^+^, CD8^+^, and CD4^+^/CD8^+^ ratio into routine clinical assessment may improve overall prognosis in pediatric MPP.

## 5 Conclusion

To sum up, the changes of pulmonary function and immune function are significantly correlated with MMP. Dynamic monitoring of FVC, FEV1, PEF, CD4+T, CD8+T and CD4+T/CD8+ can be used for disease diagnosis and prognosis evaluation.

## Data Availability

The raw data supporting the conclusions of this article will be made available by the authors, without undue reservation.
