# Supplementary material for: Dynamic changes of pulmonary function and immune function in children with mycoplasma pneumonia of different severity and their predictive value for disease prognosis: a retrospective cohort study
Source: Front Med (Lausanne). 2025 Aug 26;12:1624256. doi: 10.3389/fmed.2025.1624256 (PMC12419223; doi:10.3389/fmed.2025.1624256)
Supplement: Supplementary file 1 [file Table_1.DOCX]

Table S1. Baseline Clinical Characteristics of Participants

| **Variable** | **Control (n=75)** | **Mild MPP (n=75)** | **Moderate MPP (n=75)** | **Severe MPP (n=75)** | **P-value** |
| --- | --- | --- | --- | --- | --- |
| Age (years, mean ± SD) | 6.7 ± 2.1 | 6.8 ± 2.0 | 6.6 ± 1.9 | 6.9 ± 2.2 | 0.73 |
| Male sex (%) | 53.3% | 52.0% | 54.7% | 50.7% | 0.88 |
| BMI (kg/m², mean ± SD) | 16.2 ± 2.0 | 16.1 ± 1.8 | 16.3 ± 2.1 | 16.0 ± 2.3 | 0.76 |

Note: One-way ANOVA or Chi-square tests were used for comparisons as appropriate.
